# Microleakage of pit and fissure sealings placed after enamel conditioning with phosphoric acid or with self‐etching primers/adhesives

**DOI:** 10.1002/cre2.420

**Published:** 2021-04-07

**Authors:** Stefanie Amend, Roland Frankenberger, Christina Boutsiouki, Vanessa Scharrelmann, Julia Winter, Norbert Krämer

**Affiliations:** ^1^ Department of Pediatric Dentistry, Dental School University of Marburg and University Medical Center Giessen and Marburg, Campus Giessen Giessen Germany; ^2^ Department of Operative Dentistry, Endodontics, and Pediatric Dentistry, Dental School University of Marburg and University Medical Center Giessen and Marburg, Campus Marburg Marburg Germany

**Keywords:** etch‐and‐rinse, microleakage, pit and fissure sealing, self‐etch

## Abstract

**Background:**

It is still not fully understood what pretreatment is best for achieving maximum tightness for pit and fissure sealings (PFS).

**Aim:**

This study investigated microleakage of PFS placed after etching with phosphoric acid or after the application of self‐etching primers/adhesives (SEPA).

**Design:**

131 third molars were assigned to ten groups. In Hel‐P, Helioseal® was applied after phosphoric acid etching. In the other groups, SEPA were used (Dyr‐AP: Adper™ Prompt™ L‐Pop™, Dyract® Seal; Bea: BeautiSealant Primer and Paste; Hel‐Exp: Experimental primer, Helioseal®; Hel‐Cl: Clearfil™ SE Bond^1^, Helioseal®). Specimens were stored in distilled water at 37°C (28 days), followed by 3500 thermocycles and staining with 5% methylene blue (M) or 5% silver nitrate (S). After methylene blue staining and sectioning, microleakage was assessed light microscopically. During silver nitrate staining, specimens were dissolved by 32% HCl and remaining PFS were evaluated under a SEM.

**Results:**

Tightness, percentage of penetrated area, and maximum dye penetration were best for Hel‐P and Hel‐Cl (*p* < 0.05).

**Conclusions:**

Phosphoric acid etching of enamel and Clearfil™ SE Bond resulted in the best sealing quality. Methylene blue staining allowed the evaluation of more criteria (fissure shape, voids, sealant penetration depth) compared to silver nitrate.

## 1 INTRODUCTION

Recent systematic review shows that fissure sealing with resin‐based materials has been an effective way to reduce caries formation by 11%–51% in 2 years compared to no sealing (Ahovuo‐Saloranta et al., 2017). Pit and fissures of first permanent molars are the first to be affected by caries up to the age of 12 years, even in countries with mean DMFT <2 (Marthaler, 2004). It is this way clear that pit and fissure sealants are a very important tool in caries prevention. However, as with all resin materials, adhesion is the Achilles' ptern of long‐term preservation of sealants and loss of it leads to microleakage (Kidd, 1976). When a resin‐based material is placed, competition arises between polymerization shrinkage forces and bond strength to the subsequent dental structure (Van Meerbeek et al., 2011). If bond strength between sealant and enamel is weaker, a fracture is caused and a way through the gap is formed as the material separates itself from enamel, therefore resulting in microleakage and failure of the restoration (Kidd, 1976). A minimal degree of leakage can be tolerated and not cause a reaction. But in some cases it can become the source of postoperative pain and recurrent caries, leading to restoration failure (Kuhnisch et al., 2012).

Resin sealants exhibit the highest retention rate after 5 years (83.8%) compared to glass ionomer sealants (5.2%) (Kuhnisch et al., 2012). A disadvantage of resin sealants compared to glass ionomer cements is their technique sensitivity during clinical application, as excellent control of moisture is needed. According to their composition resin‐based sealants can be conventional resins, compomers or giomers^2^. Compomers are polyacid‐modified composites with fluoride‐releasing silicate glasses (Kuhnisch et al., 2012). An acid–base reaction takes place as compomer absorbs water, which facilitates cross‐linking structure and fluoride release. Giomers are composites with pre‐reacted glass‐ionomer fillers in their resin matrix (Ntaoutidou et al., 2018).

Pit and fissure sealing is described as a “micro‐invasive treatment,” as the conditioning of the tooth surface results in an irreversible removal of a small amount of dental hard tissue (Schwendicke et al., 2015). The outer enamel layer of permanent teeth is prismless and even when it's abraded due to mastication, the inner surface of the fissure, where sealants are placed, remains prismless (Schwendicke et al., 2015). For the conditioning of the prismless enamel layer, several treatment options have been suggested in the literature, like the application of phosphoric acid (Erdemir et al., 2014), the use of self‐etching primers/adhesives (Ntaoutidou et al., 2018), air‐abrasion (Kramer et al., 2008) or laser conditioning (Karaman et al., 2013). Etching the enamel with 35%–37% phosphoric acid for at least 30 s is the gold standard method to remove the prismless enamel surface layer prior to the application of resin‐based pit and fissure sealants. Over the past years, self‐etching primers/adhesives have been tested as pretreatment for pit and fissure sealing sacrificing the etching with phosphoric acid and rinsing with water‐spray (Ntaoutidou et al., 2018). Self‐etching primers/adhesives are supposed to condition the enamel with acidic functional groups and polymerizable acidic components (Pashley & Tay, 2001). Among their advantages, they reduce clinical steps (Perry & Rueggeberg, 2003) and the possibility of contamination of the occlusal surface with saliva, over‐drying/wetting is avoided and they can be easily used in children with limited compliance (Ntaoutidou et al., 2018). On the one hand, systematic reviews show worse adhesion of sealants placed after the application of self‐etching primers/adhesives to enamel compared to etching with phosphoric acid (Birlbauer et al., 2017; Botton et al., 2016; Pitchika et al., 2018). On the other hand, retention (Erbas Unverdi et al., 2017) and microleakage (Nahvi et al., 2018) of sealants used in combination with self‐etching primers/adhesives have been shown to be equal to sealants with prior etching. However, marginal leakage of sealants with self‐etching primers could also be due to unsuccessful adhesion at first place, as self‐etching adhesives show reduced adhesion to enamel (Van Meerbeek et al., 2011). pH of the self‐etching primer, as well as addition of functional monomers like 10‐MDP in its composition, also play an important role on the adhesive performance of self‐etching adhesives (Van Meerbeek et al., 2011).

In microleakage studies a penetrant is needed in order to mark the available areas for penetration around restorations or around sealants. The one mostly used is methylene blue (Agrawal & Shigli, 2012; Kramer et al., 2008), followed by fuchsine (Gillet et al., 2002; Hatirli et al., 2018; Heintze et al., 2008), and the more expensive silver nitrate (Heintze et al., 2008). A review by Heintze et al. (2008) showed no difference between the aforementioned substances (Heintze et al., 2008).

The aim of the present study was to compare the efficacy of a pretreatment with 37% phosphoric acid prior to pit and fissure sealing with the utilization of self‐etching primers/adhesives as conditioners. Furthermore, the results of two dye penetration tests, namely either with 5% methylene blue or with 5% silver nitrate, used for the assessment of the quality of preventive pit and fissure sealants in vitro should be checked against each other. The null hypotheses tested were: (i) different procedures of enamel conditioning have no impact on the sealing ability of pit and fissure sealants and (ii) there are no differences between the two dye penetration methods.

## 2 MATERIAL AND METHODS

### 2.1 Specimens selection and preparation

This study has followed the CRIS guidelines for in‐vitro studies as discussed in the 2014 concept note (Krithikadatta et al., 2014). The conduction of the present laboratory study was approved by the local ethics committee of the Justus‐Liebig‐University Giessen, Germany (AZ 143/09).

131 caries‐free (ICDAS II Code 0), permanent third molars were collected and stored in 0.5% chloramine‐t solution (Chloramin T Trihydrat, Carl Roth, Karlsruhe, Germany) for up to 21 days at 4°C. Occlusal surfaces were cleaned by air polishing (PROPHYflex 3, KaVo Dental, Biberach/Riss, Germany; powder: Clinpro™ Glycine Prophy Powder, 3M Oral Care, Seefeld, Germany), and they were then randomly allocated to the following experimental groups (*n* = 20): (i) 37% phosphoric gel + Helioseal® (Ivoclar Vivadent, Shaan, Liechtenstein), control group (Hel‐P), (ii) Adper™ Prompt™ L‐Pop™ (3M Oral Care) and Dyract® Seal (DENTSPLY DeTrey, Konstanz, Germany) (Dyr‐AP), (iii) BeautiSealant Primer and Paste (SHOFU Dental, Ratingen, Germany) (Bea), (iv) Experimental primer and Helioseal® (Ivoclar Vivadent) (Hel‐Exp), (v) Clearfil™ SE Bond (Kuraray Noritake Dental, Okayama, Japan) and Helioseal® (Ivoclar Vivadent) (Hel‐Cl) (Table 1). Helioseal® was tested as a conventional resin sealant, Dyract® Seal as a compomer and BeautiSealant as a giomer. Sealants were placed by a single calibrated operator and were polymerized with Bluephase lamp (Ivoclar Vivadent/light output of 1200 mW/cm^2^ ± 10%). Half of the specimens proceeded to methylene blue penetration test (M) and the other half to silver nitrate penetration test (S). Specimens were stored in distilled water at 37°C for 28 days (Incubator Typ B20, Heraeus Holding, Hanau, Germany), and were then thermocycled for 3500 cycles (+5°C and +55°C, dwell time 30 s; TCS 30, Syndicad, Munich, Germany) to simulate an artificial aging.

**TABLE 1:** Information about the materials under investigation, and their experimental procedures

Group	Material	Manufacturer	LOT Nr	Components	pH	Experimental procedure
**Hel‐P**	Pluraetch	Pluradent	T02656	37% H_3_PO_4_ in aqueous solution, thickeners, dye	0.9	Etching gel applied for 60 s, rinsed off with water spray for 10 s, air‐dried to visually control etching pattern
	Helioseal®	Ivoclar Vivadent	S38608	Bis‐GMA, DMA, TiO_2_, photoinitiator, stabilizers	‐	Sealant applied, then polymerization for 40 s
**Dyr‐AP**	Adper™ Prompt™ L‐Pop™	3M Oral Care	569,183	Liquid 1 (red cushion): MOP, Bis‐GMA, photoinitiator, stabilizers Liquid 2 (yellow cushion): water, HEMA, polyalkene acid, stabilizers	1	Adhesive applied for 15 s, gently dried with air (repeated three times with new blister each time), polymerized for 30 s
	Dyract® Seal	DENTSPLY DeTrey	1,402,000,074	Strontium‐alumino‐fluoro‐phosphor‐silicate glass, SiO_2_, ammonium salt of phosphoric acid modified methacrylate resin, carboxylic acid modified methacrylate resin, DGDMA, CQ, EDMAB, BHT	‐	Sealant applied, and then polymerized for 40 s
**Bea**	BeautiSealant Primer	SHOFU Dental	91,421	Aceton, distilled water, carboxylic acid monomer, phosphonic acid monomers and others	2.3	Primer applied for min. 10 s, gently dried with air
	BeautiSealant Paste	SHOFU Dental	91,443	S‐PRG filler based on fluoroboroaluminosilicate glass, UDMA, TEGDMA, micro fumed silica and others	‐	Sealant applied, and then polymerized for 40 s
**Hel‐Exp**	Experimental Primer	Ivoclar Vivadent	T30712	Methacrylic acid derivatives, solvents, photoinitiator, stabilizers	2	Applied for 15 s, gently dried with air, polymerized for 40 s
	Helioseal®	Ivoclar Vivadent	S38608	Bis‐GMA, DMA, TiO_2_, photoinitiator, stabilizers	‐	Sealant applied, and then polymerized for 40 s
**Hel‐Cl**	Clearfil™ SE Bond	Kuraray Noritake Dental	000122	Primer: MDP, HEMA, DMA, CQ, water, catalyst, dye Bond: Bis‐GMA, HEMA, MDP, DM, microfiller, CQ, photoinitiator, catalyst	2	Primer applied for min. 20 s, gently dried with air, polymerized for 30 s
	Helioseal®	Ivoclar Vivadent	38,608	Bis‐GMA, DMA, TiO_2_, photoinitiator, stabilizers	‐	Sealant applied, then polymerization for 40 s

Abbreviations: BHT, butylated hydroxytoluene; Bis‐GMA, bisphenol A diglycidyl methacrylate; CQ, camphorquinone; DGDMA, diethyleneglycol dimethacrylate; DMA, dimethacrylate; EDMAB, ethyl‐4‐dimethylaminobenzoate; MDP, 10‐Methacryloyloxydecyl‐Dihydrogenphosphat; MOP, methacrylate organophosphate; SiO_2_, silicon dioxide; TiO_2_, titanium dioxide.

### 2.2 Methylene blue penetration test (M)

For the conduction of methylene blue penetration test, specimens' apices were sealed with glue wax (Chemical Dental Laboratory Oppermann‐Schwedler, Bonn, Germany), and the roots were covered with acid resistant nail varnish (Manhattan, Stuttgart, Germany) to prevent a retrograde dye penetration. Followed to that, specimens were centrifuged for 5 min at 30*g* in 5% methylene blue solution (Heraeus Megafuge 8, Heraeus Holding). Upon completion, 1 mm thick slices were produced by a microtome (IsoMet™ 1000 Precision Saw, Diamond Wafering Blade Series 15LC, Buehler, ITW Test & Measurement, Dusseldorf, Germany); the blade being placed perpendicularly to the occlusal surfaces in bucco‐lingual direction.

**FIGURE 1:**
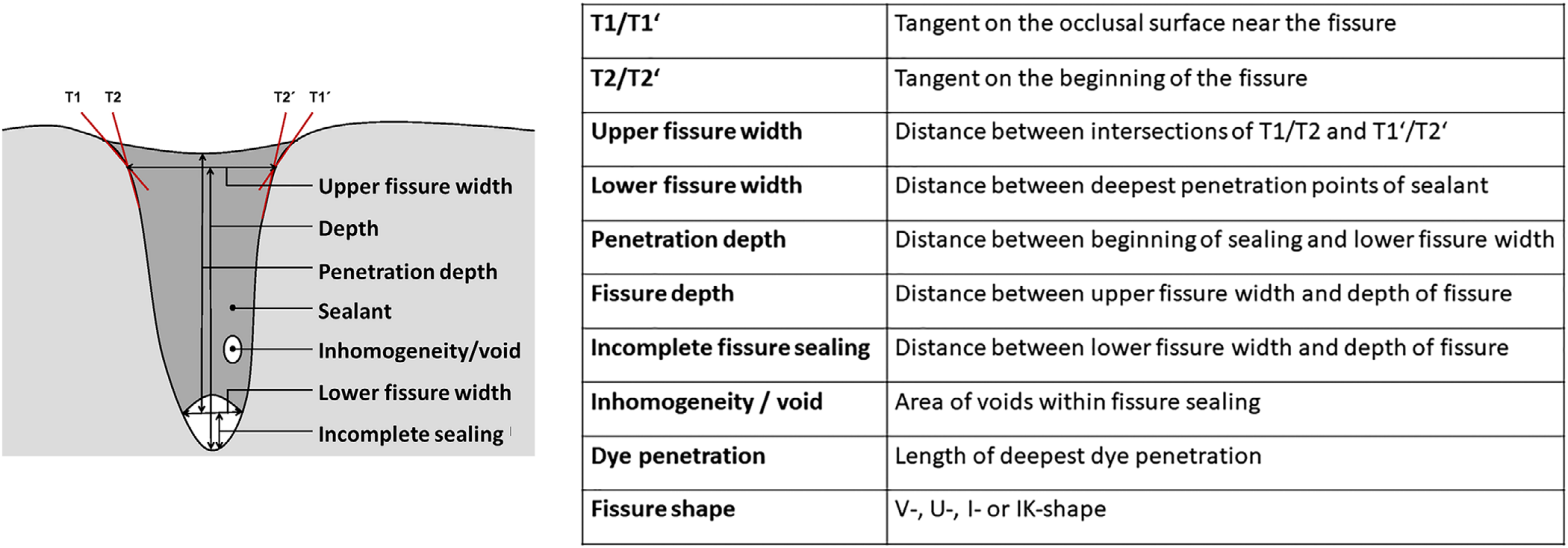
Evaluation criteria for the methylene blue penetration test

Microscopic evaluation at 40x magnification was performed with a light microscope (Nikon AZ100 M, Nikon, Tokyo, Japan; Software NIS‐Elements 4.00.01), and determination of fissure shape (V‐, U‐, I‐ and IK‐shape) followed. Assessment of the sealing quality was made upon the following criteria: upper and lower fissure width (in μm), fissure depth (in μm), sealant penetration depth into the fissure (in μm), inhomogeneity/voids within the sealant (in %), dye penetration depth (in μm) (Figure 1) (Kramer et al., 2008). Tightness was defined as the absence of dye penetration around the sealant and was calculated from the maximum percentage of dye penetration (see below). The even distribution of fissure shapes on the five different groups was tested by comparing the upper/lower fissure widths.

### 2.3 Silver nitrate penetration test (S)

Sealed teeth were stored in 5% AgNO_3_ solution for 24 h at 37°C (Incubator Typ B20, Heraeus Holding), followed by a storage in 0.5% *tert*‐Butylhydrochinon solution for 24 h at 37°C (Incubator Typ B20, Heraeus Holding) in order to reduce silver ions concentration. For detachment of the pit and fissure sealings from the occlusal surfaces, specimens were immersed in 32% HCl for 5–6 h. Finally, the remaining pit and fissure sealings were removed from the laboratory glasses (No. X655.1, 10 ml, Carl Roth), while remnants of hydrochloric acid were rinsed off with distilled water. Air drying of the pit and fissure sealings followed.

**FIGURE 2:**
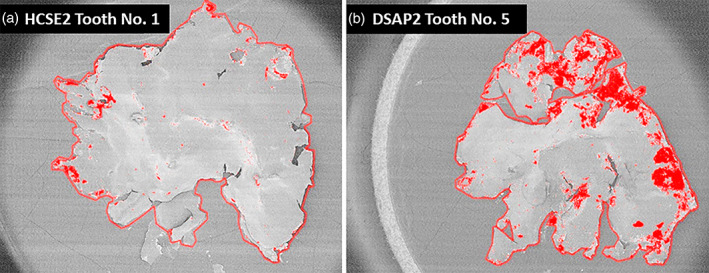
Exemplary illustration of the percentage of penetration area of the pit and fissure sealant. On image 2a, the percentage of penetrated area is small, whereas, it is high on image 2b

Evaluation was performed under SEM. After sputtering of the pit and fissure sealing with a coating of gold/palladium (BAL‐TEC SCD 500 sputter Coater, Bal‐tec, Balzers, Liechtenstein) images were obtained (ZEISS SUPRA 40 VP, Carl Zeiss, Oberkochen, Germany; acceleration voltage 15 kV), on which silver particles were displayed in white, and the pit and fissure sealings having a darker color. By means of the software AnalySIS auto (version 5.1), the overall area of the pit and fissure sealings (in μm) was calculated by using the function ROI (region of interest), and by circling the outlines of each pit and fissure sealing in a distance of 2–10 μm. Silver particles were marked in red color and percentage of silver penetration was calculated (Figure 2).

### 2.4 Comparison of methylene blue penetration test (M) and silver nitrate penetration test (S)

**FIGURE 3:**
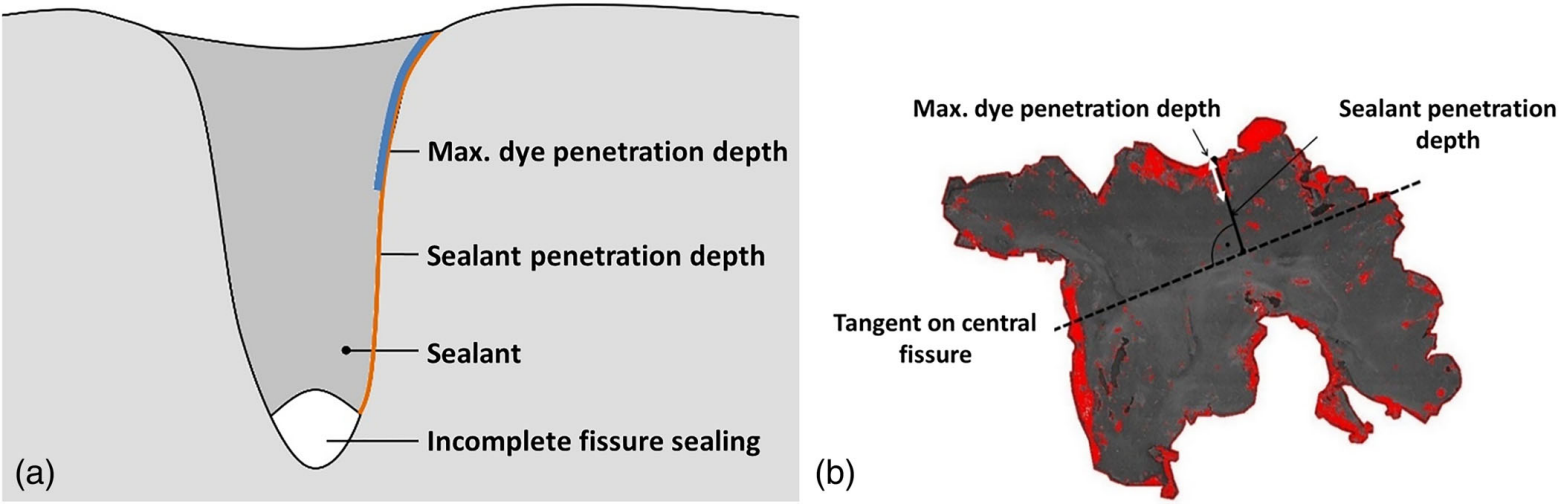
To compare the results of both dye penetration tests, the maximum dye penetration depth was assessed for specimens after the methylene blue penetration test (a), and after the silver nitrate penetration test (b)

To compare both staining methods, obtained images were re‐evaluated to assess maximum dye penetration (Figure 3). For methylene blue penetration test, the section with the highest dye penetration score was chosen. Maximum sealant penetration into the fissure and maximum methylene blue penetration around the sealant were measured to calculate the maximum percentage of dye penetration. On the images of silver nitrate dye penetration test, the percentage of dye penetration was assessed by drawing a tangent along the central fissure, measuring the line from the tangent to the outer border of the sealing in the area of maximum sealant penetration, and assessing the penetration depth of the sealant along that line (Figure 3).

### 2.5 Statistical analysis

Statistical analysis was performed with SPSS 26.0 (IBM Statistics, Armonk, NY, USA). Normal distribution was checked with Kolmogorov–Smirnov test. Medians and interquartile ranges (IQR) were calculated for the evaluation criteria of the methylene blue penetration test (tightness, homogeneity, sealant penetration depth [in %], maximum dye penetration depth, sealant penetration depth [in nm]) and of the silver nitrate penetration test (penetrated area by silver nitrate, total area of sealed fissure, maximum dye penetration depth, sealant penetration depth [in nm]). Non‐parametric tests (Kruskal–Wallis) combined with post‐hoc tests and Bonferroni correction for multiple comparisons were used to show statistically significant differences between the sealant groups. Differences between the two penetration tests were studied with Mann–Whitney U tests for pairwise comparisons. The significance level was set at *p* < 0.05.

## 3 RESULTS

No significant differences were noted between the shape of the fissures among the groups (*p* = 0.104, Kruskal–Wallis), showing that fissure forms were equally distributed.

### 3.1 Methylene blue penetration test (M)

Tightness was significantly higher for control group compared to Dyr‐AP‐M (*p* < 0.001, Bonferroni) and Bea‐M (*p* = 0.009, Bonferroni). It was lower for Dyr‐AP‐M compared to Hel‐Exp‐M (*p* = 0.023, Bonferroni), or Dyr‐AP‐M compared to Hel‐Cl‐M (*p* = 0.001, Bonferroni). Bea‐M also showed significantly lower tightness compared to Hel‐Cl‐M (*p* = 0.021, Bonferroni) (Table 2).

**TABLE 2:** Medians [IQR] of parameters evaluated at methylene blue penetration test.

	Tightness (in %)	Homogeneity (in %)	Sealant penetration depth (in %)
**Hel‐P‐M**	100 [100‐100]^B^	93 [83‐100]^A,B^	78 [67‐92]^A^
**Dyr‐AP‐M**	63 [54‐74]^A^	100 [100‐100]^A^	99 [93‐100]^B,C^
**Bea‐M**	71 [39‐86]^A,C^	86 [71‐86]^B^	79 [69‐85]^A^
**Hel‐Exp‐M**	100 [83‐100]^B,C^	85 [83‐100]^A,B^	85 [81‐92]^A,C^
**Hel‐Cl‐M**	100 [100‐100]^B^	93 [82‐100]^A,B^	95 [90‐100]^B,C^

*Note*: Different upper case superscript letters represent statistically significant differences between the groups for each evaluation parameter of the methylene blue penetration test (Kruskal‐Wallis, Bonferroni correction, *p* < 0.05).

Presence of internal voids (homogeneity) in the sealants was minimum, albeit with statistical difference among the materials tested (*p* = 0.008, Kruskal–Wallis). Compomer (Dyr‐AP‐M) showed no internal voids presenting a significantly higher homogeneity than Bea‐M (*p* = 0.003, Bonferroni). Conventional resin (Hel‐P‐M, Hel‐Exp‐M, Hel‐Cl‐M) showed medians of 85%–93% for void‐free sealings (Table 2).

Sealant penetration depth (in %) was significantly higher for compomer (Dyr‐AP‐M) compared to giomer (Bea‐M; *p* = 0.002, Bonferroni) and control group (Hel‐P‐M; *p* = 0.004, Bonferroni). Conventional composite with phosphoric acid etching (Hel‐P‐M) showed worse sealant penetration depth (in %) than the same material with a self‐etching adhesive (Hel‐Cl‐M) (*p* = 0.034, Bonferroni). Moreover, specimens sealed with Hel‐Cl‐M exhibited significantly higher sealant penetration depths than Bea‐M (*p* = 0.019, Bonferroni) (Table 2).

Maximum penetration depth (in %) of methylene blue was significantly lower for the control group with the separate etching step (Hel‐P‐M) compared to Dyr‐AP‐M (*p* = 0.011, Bonferroni) and Bea‐M (*p* = 0.030, Bonferroni). Additionally, Hel‐Cl‐M showed significantly lower dye penetration opposed to Dyr‐AP‐M (*p* = 0.016, Bonferroni) and to Bea‐M (*p* = 0.041, Bonferroni) (Table 4).

### 3.2 Silver nitrate penetration test (S)

Maximum silver nitrate penetration depth (in %) was increased for Dyr‐AP‐S compared to Hel‐Cl‐S (*p* = 0.013, Bonferroni) (Table 4). The percentage of penetrated area by silver nitrate was significantly higher for the groups Dyr‐AP‐S and Bea‐S in comparison to Hel‐P‐S and Hel‐Cl‐S (*p* < 0.05, Bonferroni) (Table 3). Statistically significant differences regarding the total area of the sealed fissure were observed among groups (Kruskal–Wallis, *p* < 0.001), with Dyr‐AP‐S and Bea‐S having a higher amount of sealant applied to the pit and fissure system than the group Hel‐P‐S (*p* < 0.05, Bonferroni) (Table 3). Additionally, the total area of the sealed fissure was higher for the giomer group (Bea‐S) compared to the resin composite groups applied with self‐etching primers/adhesives (Hel‐Exp‐S: *p* = 0.043, Hel‐Cl‐S: *p* = 0.009, Bonferroni).

**TABLE 3:** Medians [IQR] of parameters evaluated at silver nitrate penetration test.

	Penetrated area by silver nitrate (in %)	Total area of sealed fissure (in pixel)
**Hel‐P‐S**	2.5 [2.2‐3.6]^A^	548768 [478536‐613594]^A^
**Dyr‐AP‐S**	7.4 [6.6‐9.0]^B,C^	1564063 [1215936‐2428156]^B,C^
**Bea‐S**	8.5 [6.6‐10.1]^B,C^	2071117 [1765240‐2547224]^B^
**Hel‐Exp‐S**	3.3 [2.7‐7.5]^A,C^	738311 [478882‐1820356]^A,C^
**Hel‐Cl‐S**	3.2 [2.6‐4.0]^A^	745916 [595831‐939715]^A,C^

*Note*: Different upper case superscript letters represent statistically significant differences between the groups for each evaluation parameter of the silver nitrate penetration test (Kruskal‐Wallis, Bonferroni correction, *p* < 0.05).

### 3.3 Comparison between the two methods

Parameters “maximum dye penetration depth (in %)” and “sealant penetration depth (in nm)” were compared for the two penetration methods tested (Table 4). Significant differences were noted between the two penetration tests regarding maximum dye penetration depth, for control group (*p* = 0.002, Mann Whitney U), Dyr‐AP (*p* = 0.043, Mann Whitney U) and Hel‐Cl (*p* = 0.002, Mann Whitney U), as the silver nitrate penetration test (S) showed significantly higher percentages of dye penetration than the methylene blue penetration test (M). Control group (*p* < 0.001, Mann Whitney U), Hel‐Exp (*p* = 0.001, Mann Whitney U) and Hel‐Cl (*p* < 0.001, Mann Whitney U) showed significantly different values for sealant penetration depth between the two tested methods. Again, the silver nitrate penetration test (S) showed higher values than the methylene blue penetration test (M).

**TABLE 4:** Medians [IQR] of dye and sealant penetration depth for both tests.

	Maximum dye penetration depth (in %)	Sealant penetration depth (in nm)
**Hel‐P‐M**	0.0 [0.0‐0.0]^A,a^	0.0 [0.0‐0.0]^A,a^
**Dyr‐AP‐M**	21.0 [12.3‐23.9]^B,C,c^	1632.4 [1310.8‐1738.5]^B^
**Bea‐M**	18.6 [4.3‐31.4]^B,C^	1410.0 [1161.3‐1820.3]^B,C^
**Hel‐Exp‐M**	0.0 [0.0‐32.8]^A,C^	0.0 [0.0‐1104.0]^A,C,c^
**Hel‐Cl‐M**	0.0 [0.0‐0.0]^A,e^	0.0 [0.0‐0.0]^A,e^
**Hel‐P‐S**	12.0 [8.5‐23.2]^A,B,b^	1849.6 [797.4‐2968.0]^b^
**Dyr‐AP‐S**	25.3 [20.1‐42.7]^B,d^	1892.5 [1607.0‐2733.2]
**Bea‐S**	21.1 [17.4‐36.5]^A,B^	1746.6 [1332.6‐2900.3]
**Hel‐Exp‐S**	14.8 [9.4‐30.5]^A,B^	3168.5 [1078.3‐3495.1]^d^
**Hel‐Cl‐S**	13.2 [7.0‐19.2]^A,f^	1802.8 [941.5‐2907.7]^f^

*Note*: Different upper case superscript letters represent statistically significant differences between the groups for each penetration test (methylene blue, silver nitrate; Kruskal‐Wallis, Bonferroni correction, *p* < 0.05). Statistically significant differences between the two penetration tests for each pit and fissure sealant group are marked with distinct lower case superscript letters (Mann‐Whitney‐Test, *p* < 0.05). M corresponding to methylene blue penetration test and S to silver nitrate penetration test.

## 4 DISCUSSION

The present study aimed to compare the use of phosphoric acid versus self‐etching primers/adhesives as enamel pretreatment prior to sealants application. Three different resin materials were used as pit and fissure sealants, Helioseal as a conventional resin‐based sealant, the compomer Dyract Seal, the giomer BeautiSealant; and two different dye penetration methods, methylene blue and silver nitrate penetration test, were compared.

Methylene blue penetration test is a reliable method for the evaluation of microleakage adjacent to pit and fissure sealants and has been used in several studies (Gillet et al., 2002). Further advantages of using the methylene blue penetration test are that the dye is not expensive and it is easy to use. On the basis of the microscopic images, many parameters may be measured and specimens can be investigated microscopically (Brocklehurst et al., 1992). The time‐consuming specimen preparation and measurements together with the toxicity of methylene blue (acute toxicity after oral intake, hazardous to the aquatic environment) are regarded as disadvantages (Van Meerbeek et al., 2011). For silver nitrate penetration test, the specimen preparation and evaluation may be completed faster. During microscopic evaluation, the entire pit and fissure sealing may be investigated, as dental hard tissues have been dissolved before by storage in hydrochloric acid. Compared to methylene blue penetration test, less parameters are measurable because only sealant material remains for evaluation after dissolution of the specimens' dental hard tissue. All in all, specimens stained with methylene blue allow for the evaluation of additional parameters, as specimens are sectioned with a microtome enabling the assessment of the fissure shape, the number of voids, and the sealant penetration depth, as it was done in the present study. Moreover, the time‐consuming and expensive manufacturing of the solutions for silver nitrate penetration test combined with the possible hazards of the chemicals (etching/irritation of eyes/skin, hazardous to the aquatic environment) are disadvantageous for this penetration test (Van Meerbeek et al., 2011). Both penetration tests showed similar % maximum dye penetration within the groups, with the silver nitrate test exhibiting higher percentages, however, significant differences were material dependent (Table 4). A possible explanation for this could be the fact that with silver nitrate penetration test the whole sealant surface is evaluated for dye penetration, while with methylene blue, evaluation is performed at a certain number of cuts, therefore at a limited percentage of the sealed area. Therefore, null hypothesis ii was rejected.

Regarding specimen preparation, prior to the sealant application it is recommended to clean the occlusal surface either with a low‐speed rotating bristle brush combined with pumice slurry or by air polishing, the later was used in the present study. Air polishing was chosen since it has been shown to increase bond strength of sealants and to enhance sealant penetration depth in vitro *(*Brocklehurst et al., *1992* *)*. Despite the fact that fissures demonstrate a variety of shapes (Nagano, 1961), homogenous distribution of specimens along the experimental groups allowed for an unbiased evaluation.

Thermocycling was performed in order to simulate the thermal changes which take place in the oral environment. In accordance with literature, temperature changed alternatively from 5 to 55°C (Heintze et al., 2008). Despite the fact that some published studies show no differences when thermocycling was performed, it was chosen in order to comply with the most of microleakage literature.

After the introduction of phosphoric acid conditioning by *Buonocore* in 1955, enamel etching has been regarded as the gold standard in order to achieve adhesion via micromechanical retention of resin composites and therefore resin pit and fissure sealants (Buonocore, 1955). The application of phosphoric acid exposes prismatic enamel, creates microporosities into which resin‐based sealants penetrate and once polymerized, resinous tags and mechanical anchoring are produced (Gwinnett, 1973). According to a recently published systematic review and meta‐analysis, phosphoric acid etching combined with the use of resin‐based sealants exhibited the best clinical long‐term performance in terms of favorable retention rates. All in all, the longevity of sealants placed in combination with primers/adhesives was substandard, though depending on the type of primer/adhesive. The results of the present study confirm the good pit and fissure sealing ability after phosphoric acid etching of enamel, as Hel‐P with separate phosphoric acid etching demonstrated lower % maximum dye penetration depth compared to most of the other groups, with both evaluation methods (Table 4). Therefore, null hypothesis i was partially rejected in this respect. On the other hand, self‐etching primers/adhesives with acidic methacrylate monomers that do not have to be rinsed off have shown to reduce the time needed for pit and fissure sealing by 1/3 compared to etching with phosphoric acid (1.8 min vs. 3.1 min). This is advantageous in pediatric dentistry due to the limited compliance of children during treatment (Ntaoutidou et al., 2018).

Considering pH‐values of the materials used in the present study, 37% H_3_PO_4_ in aqueous solution had a pH of 0.9, whereas the self‐etching primers/adhesives were milder with pH values ranging from 1 to 2.3 (Table 1). Literature comparing phosphoric acid etching with self‐etching primers/adhesives shows worse behavior of self‐etching primers. An in vitro study by Kanemura et al. (1999) comparing the application of either phosphoric acid or self‐etching primers to intact enamel showed that self‐etching primers demineralized the enamel insufficiently and resulted in swallower adhesive penetration, shorter resin tags as well as lower bond strength (Kanemura et al., 1999). Short resin tags were also found by Torii et al. (2002) after the application of self‐etching adhesives on ground bovine enamel, although tensile bond strength was comparable to etch‐and‐rinse adhesives (Torii et al., 2002). As a consequence, the inability of self‐etching primers/adhesives to etch and penetrate deep into intact enamel of the occlusal surface may be a reason for the increased microleakage observed for Dry‐AP and Bea in both dye penetration methods (Table 4). This finding is also in agreement with the microleakage study by Perry and Rueggeberg (2003).

In group Dyr‐AP, the self‐etching adhesive Adper™ Prompt™ L‐Pop™ was combined with the compomer Dyract® Seal. The application of Adper™ Prompt™ L‐Pop™ was conducted in three layers, as multi‐coating has been shown to increase microtensile bond strength (Frankenberger et al., 2001). Furthermore, the combination of this adhesive with a compomer increased bond strength in vitro (Frankenberger et al., 2001). For this reason, Dyract® Seal was chosen as pit and fissure sealant in this group. A demineralization pattern similar to the one obtained after phosphoric acid conditioning was caused when Promp™ L‐Pop™, with a pH of 1, was applied on prismless enamel. Nevertheless, the use of this self‐etching adhesive resulted in significantly lower microtensile bond strength to aprismatic enamel compared to phosphoric acid etching. A clinical trial by Yilmaz et al. (2010) revealed a significantly lower retention rate for Dyract® Seal applied with a self‐etching primer/adhesive compared to resin‐based sealants and ormocers after 24 months (Yilmaz et al., 2010). A meta‐analysis by *Kühnisch* et al. confirmed an unfavorable retention rate of 17.9% (95%‐CI: 8.2%–58.0%) after 3 years, and 3.8% (95%‐CI: 0.2%–31.8%) after 5 years for compomers used as pit and fissure sealants. In the present study, the tightness of sealings in group Dyr‐AP (63% [54%–74%]) was low, though the sealant penetration depth was high (99% [93%–100%]) indicating an insufficient adaption of this dental material to aprismatic enamel (Table 2).

Giomer BeautiSealant, a resin‐based sealing material additionally containing inorganic surface pre‐reacted glass ionomer cement (S‐PRG) fillers based on fluoroboroaluminosilicate glass (Ntaoutidou et al., 2018), was applied in group Bea. It is used in combination with the self‐etching BeautiSealant Primer having a mild pH of 2.3. Ntaoutidou et al. (2018) assessed a total retention rate of as low as 6.9% for Beautisealant after 18 months in a randomized controlled clinical trial. A possible explanation was the limited demineralization potential of its self‐etching primer (Ntaoutidou et al., 2018), which may also be an explanation for the higher microleakage of BeautiSealant (Table 4).

Different pretreatments seemed to have an impact on the penetration depth of the Bis‐GMA‐based sealant Helioseal® into the fissure system. The use of Helioseal® in combination with the self‐etching adhesive Clearfil™ SE Bond (Hel‐Cl) resulted in the highest sealant penetration depth into the fissure (95% [90%–100%), followed by the group Hel‐Exp being pretreated with an Experimental primer (85% [81%–92%]), and a lower sealant penetration depth in the group etched with phosphoric acid (78% [67%–92%]). This can be explained by alteration of the contact angle of the occlusal enamel surface after the application of different acids or primers, thus altering the ability of the sealant to flow and spread into the fissure. The tightness of fissures sealed with Helioseal® was high, independently of the pretreatment (phosphoric acid vs. self‐etching primer/adhesive) used in this in vitro study (Table 2), showing that the material tightly seals the pit and fissure system.

## 5 CONCLUSION

Within the limitations of this in vitro study, enamel etching with 37% phosphoric acid, or application of Clearfil™ SE Bond as a self‐etching adhesive prior to sealing pit and fissures with resin‐based sealants, resulted in the lowest microleakage, and therefore tightest sealing.

The use of the methylene blue penetration test facilitated the evaluation of more parameters compared to the silver nitrate penetration test and was less expensive. Therefore, methylene blue penetration test may be recommended for microleakage studies in the laboratory.

## CONFLICT OF INTEREST

SA, CB, VS, and JW have no conflict of interest. RF and NK got research grants and speaker honorarium from Ivoclar and Dentsply.

## AUTHOR CONTRIBUTIONS

Stefanie Amend wrote the paper. Roland Frankenberger and Norbert Krämer conceived the ideas and methodology. Christina Boutsiouki did literature work. Vanessa Scharrelmann did the experiments. Julia Winter did proofreading and assistance with literature work.

## Data Availability

Data available.

## Endnote

^2^
Compomer and Giomer sealings showed an inferior performance.
